# From [^11^C]CO_2_ to [^11^C]amides: a rapid one-pot synthesis *via* the Mitsunobu reaction†
†Electronic supplementary information (ESI) available: Experimental details and NMR spectra. See DOI: 10.1039/c7cc01407d


**DOI:** 10.1039/c7cc01407d

**Published:** 2017-04-27

**Authors:** S. Bongarzone, A. Runser, C. Taddei, A. K. Haji Dheere, A. D. Gee

**Affiliations:** a Division of Imaging Sciences and Biomedical Engineering , King's College London , King's Health Partners , St. Thomas' Hospital , London , SE1 7EH , UK . Email: antony.gee@kcl.ac.uk ; Email: salvatore.bongarzone@kcl.ac.uk

## Abstract

A novel amide synthesis methodology is described using amines, CO_2_ and Grignard reagents and Mitsunobu reagents. The method was applied to carbon-11 radiochemistry to label amides using cyclotron-produced [^11^C]CO_2_. The synthetic utility of the one-pot labelling methodology was demonstrated by producing [^11^C]melatonin. The incorporation of [^11^C]CO_2_ into [^11^C]melatonin was 36% – determined by radioHPLC 2 min post [^11^C]CO_2_ delivery.

## Abstract 2

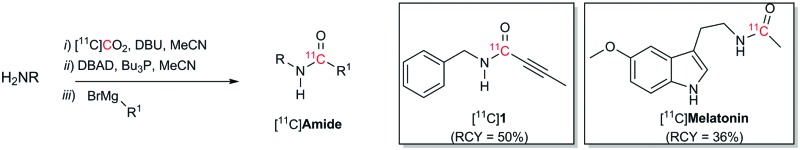
Radiosynthesis of [^11^C]amides *via* the Mitsunobu reaction.

## 

Positron emission tomography (PET) is a non-invasive imaging technology used for *in vivo* molecular imaging.^1^ Carbon-11 (^11^C) is a short-lived positron-emitting radionuclide widely used in labelling radiopharmaceuticals for medical research, diagnosis, staging and monitoring treatments. Its physical and nuclear characteristics (radioactive half-life = 20.4 min) and its orthology with carbon-12 make ^11^C an attractive radionuclide for labelling and molecular imaging. The development of rapid and reliable chemical methods for incorporating carbon-11 into organic molecules is required to expand the repertoire of available radiotracers for *in vivo* imaging studies. Cyclotron-produced [^11^C]CO_2_ is obtained by the proton bombardment of nitrogen-14 gas *via* the ^14^N(p,α)^11^C nuclear reaction. Because of its low reactivity and solubility in organic solvents, [^11^C]CO_2_ is usually converted into a more reactive secondary precursor (*e.g.* [^11^C]iodomethane^2^) in order to assemble a radiolabelled molecule of interest.^3^ The production of a secondary precursor results in significant losses due to non-quantitative yields and additional processing times. Methods for the direct incorporation of [^11^C]CO_2_ into molecules of interest would therefore have significant advantages over traditional carbon-11 labelling methodologies, since the processing times and losses due to technical handling could be minimised.

Significant efforts have been made to improve the solubility of [^11^C]CO_2_ in organic solvents, resulting in two approaches for the fixation and trapping of [^11^C]CO_2_:^4^ (1) trapping of [^11^C]CO_2_ using highly reactive organometallic reagents, such as Grignard or organolithium reagents to produce [^11^C]carboxylic acids and derivatives (*e.g.* acid chlorides);^4^ (2) utilisation of trapping agents such as 1,8-diazabicyclo[5.4.0]undec-7-ene (DBU)^5^ and 2-tertbutylimino-2-diethylamino-1,3-dimethyl-perhydro-1,3,2-diazaphosphorine (BEMP)^6^ to form a labile bond with [^11^C]CO_2_. These approaches have been applied to radiolabel [^11^C]carboxylic acids,^7^ [^11^C]amides,^8^ [^11^C]amines,^9^ [^11^C]acyl chlorides,^10^ [^11^C]ureas,^5^,^11^ [^11^C]carbamates^6^,^12^ and [^11^C]isocyanates.^13^

Current methods for the preparation of ^11^C-labelled amides utilise the coupling between [^11^C]carboxylic acids and amines at high temperatures,^8^ assisted by microwave irradiation^14^ or through activation to [^11^C]acyl chlorides using thionyl choride^15^ or phthaloyl chloride^16^ (Scheme 1). These methodologies often lead to low molar radioactivities since the reagents used readily react with atmospheric CO_2_ and have a synthesis time ranging from 5–35 minutes.^8^,^14^–^16^ In order to minimise this isotopic dilution, extreme efforts are required to control reagent stoichiometry, stability and exclusion of moisture and atmospheric CO_2_ from the reagents and reaction system.

**Scheme 1:**
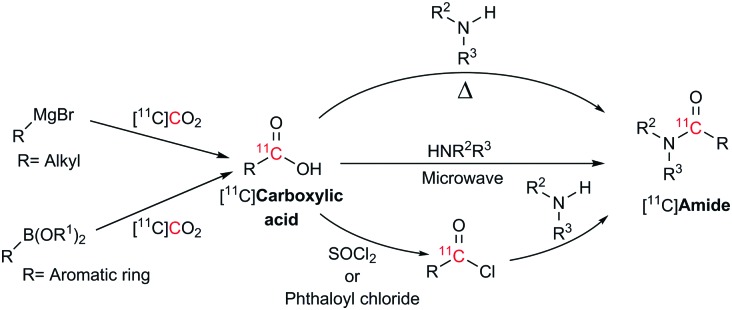
Different approaches available for the preparation of [^11^C]amides using Grignard reagents or boronic esters.

Boronic esters have higher stability to air and moisture compared with Grignard reagents and are able to react with [^11^C]CO_2_ to form [^11^C]amides in two steps within 10–15 min from [^11^C]CO_2_. However, this method is limited to the production of aromatic [^11^C]amides (R = Ar, Scheme 1).^7^

To address the shortcomings of current [^11^C]amide labelling methodologies, we sought to develop an efficient method for producing [^11^C]amides in short synthesis times, high molar radioactivities and with applicability to a range of amide derivatives bearing alkyl and aryl groups on R and R^1^ positions (Scheme 2). The synthetic strategy was inspired by our previous work on the labelling of [^11^C]ureas,^5^ which involved: (1) reaction of an amine with [^11^C]CO_2_ to form a [^11^C]carbamate anion in the presence of DBU and acetonitrile (MeCN) as a solvent (Scheme 2); (2) conversion from [^11^C]carbamate anion to an [^11^C]isocyanate or an [^11^C]oxyphosphonium intermediate using Mitsunobu reagents (tri-*n*-butyl phosphine and di-*tert*-butyl azodicarboxylate, Bu_3_P and DBAD); (3) reaction of the [^11^C]isocyanate intermediate with another molecule of amine to yield the corresponding [^11^C]urea derivative. Based on this pathway, it was hypothesised that the synthesis of [^11^C]amides might be achieved by coupling the intermediate [^11^C]isocyanate with a Grignard reagent. To test this hypothesis, the formation of amides *via* the Mitsunobu reaction was tested using both macroscopic amounts of non-radioactive CO_2_ and tracer amounts of radiolabelled [^11^C]CO_2_.

**Scheme 2:**
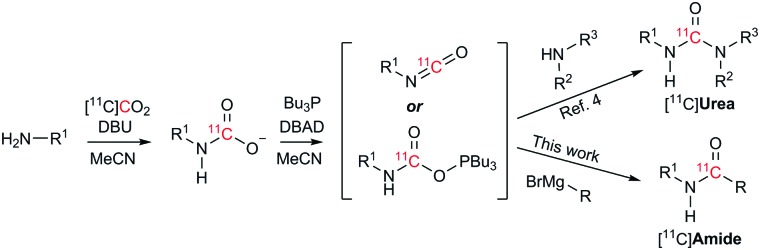
Radiosynthetic approach to radiolabelled [^11^C]ureas^5^ and [^11^C]amides (this work) from cyclotron-produced [^11^C]CO_2_.

The synthesis of **1A** was chosen as model reaction (Table 1). Initial experiments were performed adapting previously established conditions for the synthesis of urea derivatives.^17^ CO_2_ was bubbled into a solution of benzylamine (**1**) and DBU in MeCN at room temperature (r.t.) for 40 min. DBU facilitates the formation and stabilisation of the carbamate anion. Mitsunobu reagents were subsequently added and the reaction stirred for 10 min before adding an excess of 1-propynylmagnesium bromide (**A** – as a 0.5 M solution in THF, 7.2 equiv.). The reaction was quenched after 30 min and the conversion to compound **1A** was evaluated. Using the reaction conditions previously used for the synthesis of ureas, led to no observable quantities of **1A** (Table 1, entry 1). Increasing the concentration of Mitsunobu reagents or DBU had no beneficial effect on the reaction yield (Table 1, entries 2 and 3). As next step, lowering the amount of DBU from 0.1 to 0.05 equivalents different Mitsunobu reagent concentrations were tested (Table 1, entries 4–7). **1A** was obtained in 46% and 44% yield using 2 and 3.8 equivalents of Mitsunobu reagent, respectively (Table 1, entries 5 and 6). Using an equimolar or an excess of Mitsunobu reagents did not yield **1A** (Fig. S4, ESI†).

**Table 1:** Optimization of substrates and reaction conditions for the synthesis of **1A**

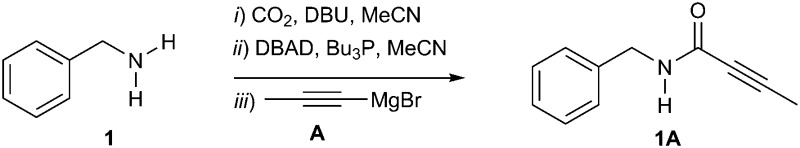				
Entry^*a*^	DBU (equiv.)	DBAD (equiv.)	Bu_3_P (equiv.)	Yield of **1A**^*b*^ (%)
1	0.1	2	2	0
2	0.1	3.8	3.8	0
3	0.2	3.8	3.8	0
4	0.05	1	1	0
5	0.05	2	2	46 ± 8^*c*^
6	0.05	3.8	3.8	44^*d*^
7	0.05	7.2	7.2	0

^*a*^Reaction conditions: CO_2_ was bubbled in a solution of **1** (138.6 μmol, 1.0 equiv.), DBU (0.05–0.2 equiv.) in MeCN (1 mL), r.t. for 40 min. Mitsunobu reagents (7.2–1 equiv.) in MeCN (0.5 mL) were added and the solution stirred for 10 min. **A** (7.2 equiv. of a 0.5 M solution in THF) was added and quenched after 30 min.

^*b*^Yield of isolated **1A** calculated from compound **1**.

^*c*^ *N* = 4.

^*d*^ *N* = 1.

The synthesis of primary, secondary and aromatic amides was explored to test the scope of the reaction (Table 2). When using the secondary amine *N*-methylbenzylamine (**2**), amide **2A** was not obtained (Table 2). The poorly nucleophilic aromatic amine (**3**) did not form the target product, probably due to its inability to form an isocyanate as reported previously.^18^ The activated aromatic amine (**4**) however gave **4A** in good yield (37%, Table 2) due to its ability to form an isocyanate intermediate in agreement with the results obtained by others.^18^

**Table 2:** Synthesis of **1A**, **4A**, **1B**, **1C**, **5C** and **6C**

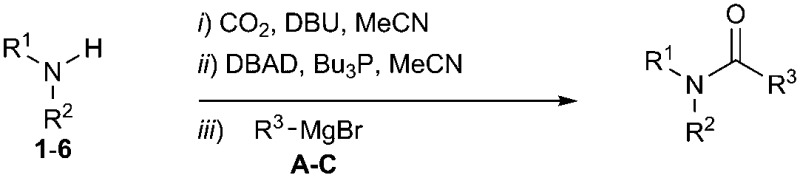				
Amine	Grignard reagent	Solvent	Product	Yield (%)
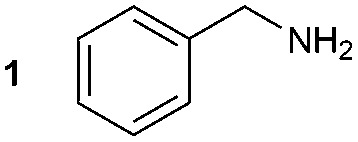	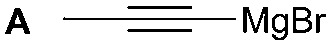	MeCN	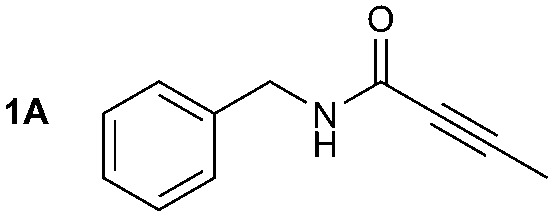	46
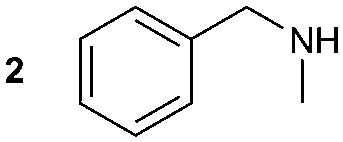	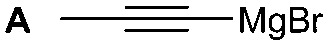	MeCN	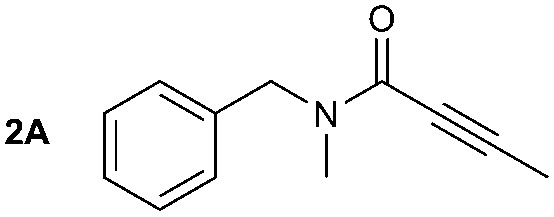	0
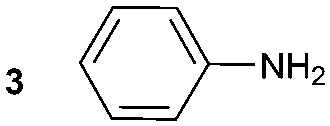	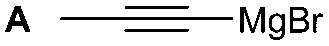	MeCN	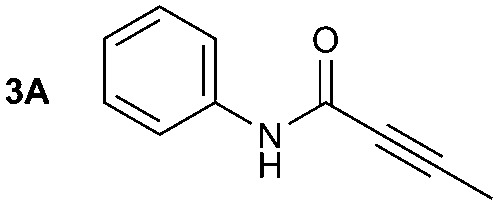	0
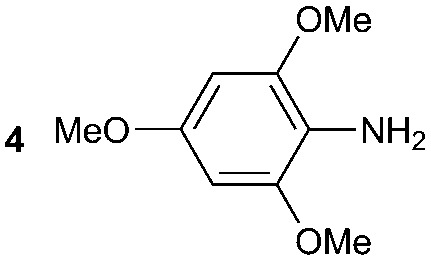	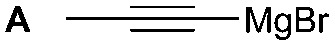	MeCN	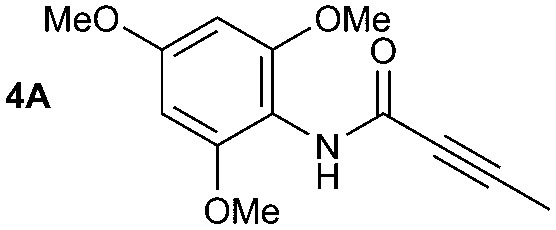	37
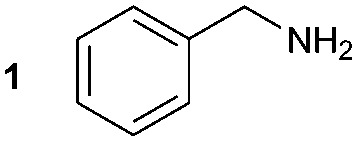	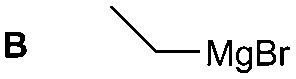	MeCN	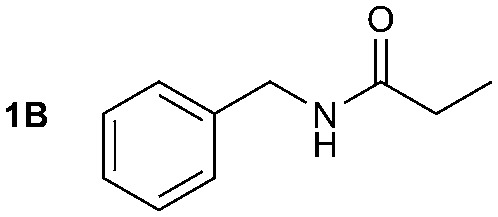	57
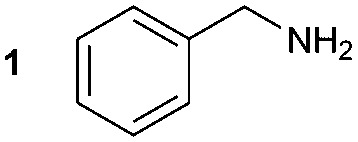	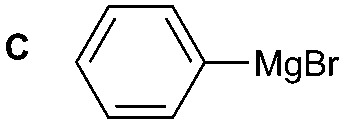	Et_2_O	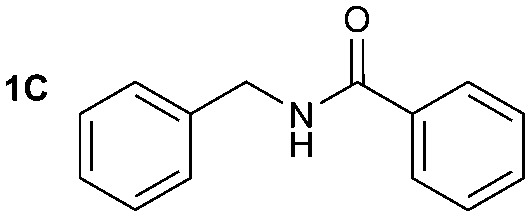	28
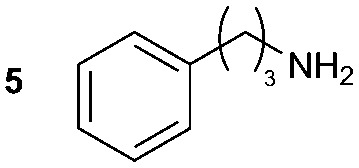	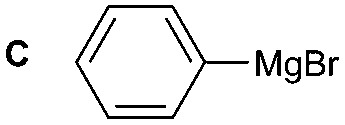	Et_2_O	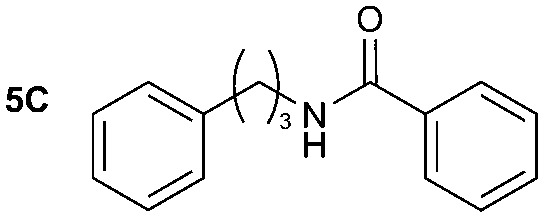	19
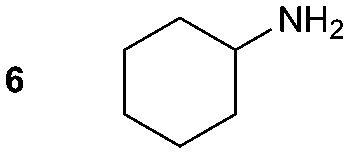	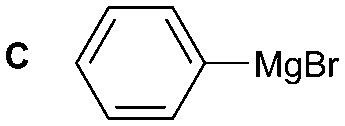	Et_2_O	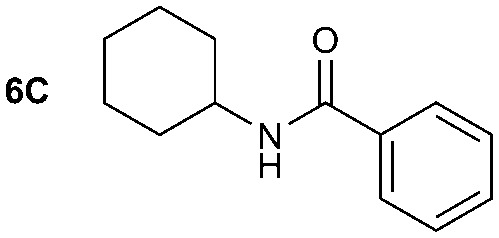	5

The reactivity of different Grignard reagents in MeCN or Et_2_O was also investigated. Reaction of **1** with ethylmagnesium bromide (**B** – as a 1.0 M solution in THF, 7.2 equiv.) in MeCN gave **1B** in a good yield (57%, Table 2), whereas when using phenylmagnesium bromide (**C** – as a 1.0 M solution in THF, 7.2 equiv.) in MeCN, the target amide was not obtained. Amide formation between isocyanates and bulky Grignard reagents such as **C** has been successfully observed using Et_2_O as solvent at 0 °C.^19^ Encouragingly, substituting the MeCN with diethyl ether (Et_2_O) under these conditions we obtained **1C**, **5C** and **6C** in yields of 5–28% (Table 2). These results indicate that primary and activated aromatic amines are able to form isocyanate intermediates and subsequently react with a broad range of Grignard reagents forming amides.

Our amide preparation methodology developed using non-radioactive CO_2_ was subsequently applied to the synthesis of [^11^C]amides using [^11^C]CO_2_. Translating the reaction conditions from synthetic chemistry to carbon-11 chemistry, we were unable to obtain [^11^C]**1A** (Table 3, entry 1), with only [^11^C]dibenzylurea present as a by-product. The solution trapped the cyclotron-produced [^11^C]CO_2_ in high efficiency (98%). As the [^11^C]CO_2_ chemistry is performed on a nano-molar scale the [^11^C]amide reaction conditions were further investigated. In an effort to reduce the amount of [^11^C]dibenzylurea by-product and optimise the radiochemical yield (RCY) of [^11^C]**1A**, the influence of changing reagent concentrations (amine, DBU and Mitsunobu reagents), temperature and solvent were studied.

**Table 3:** Radiosynthesis of [^11^C]**1A**

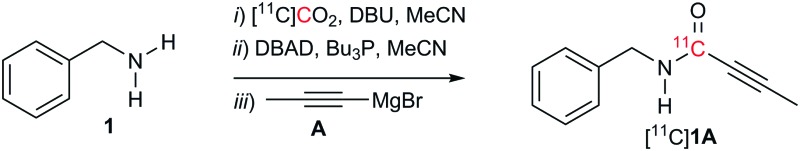				
Entry^*a*^	DBU (equiv.)	Mitsunobu reagents (equiv.)	*T* (°C)	RCY of [^11^C]**1A**^*b*^ (%)
1^*c*^	0.05	2	20	0^*g*^
2^*c*^	1	1	20	2^*f*^
3^*d*^	1	1	20	5^*f*^
4^*d*^	2.5	1	20	4^*f*^
5^*d*^	5	1	20	8^*f*^
6^*d*^	10	1	20	9.5 ± 7^*g*^
7^*d*^	10	1	30	10 ± 2^*g*^
8^*d*^	10	1	40	20 ± 4^*g*^
9^*d*^	10	1	50	18 ± 7^*g*^
10^*d*^	10	1	80	27 ± 8^*g*^
11^*d*^	10	2	20	10 ± 4^*g*^
12^*d*^	10	2	50	36 ± 4^*g*^
13^*d*^	10	2	80	45 ± 5^*g*^
14^*d*^	10	4	80	50 ± 9^*h*^
15^*d*^	10	8	80	50 ± 5^*h*^
16^*d*^	20	2	80	28 ± 5^*g*^
17^*d*^	30	4	80	30 ± 2^*g*^
18^*e*^	10	8	80	0^*f*^

^*a*^Reaction conditions: [^11^C]CO_2_ was bubbled in a solution of **1** (32 μmol, 1 equiv.), DBU (0.05–30 equiv.) in MeCN (160 μL) at room temperature. Then, the reaction mixture was heated (20–80 °C) for 30 seconds. Mitsunobu reagents (1–8 equiv.) in MeCN (100 μL) were added and stirred for 10 s. **A** (8 equiv. of a 0.5 M solution in THF) was added and quenching after 1 min.

^*b*^RCY determined by radio-HPLC not decay-corrected.

^*c*^138.5 μmol of **1**.

^*d*^32 μmol of **1**.

^*e*^32 μmol of **1** in DMF.

^*f*^ *N* = 1.

^*g*^ *N* = 2.

^*h*^ *N* = 3.

Initial experiments were performed with higher amounts of DBU. A low RCY^20^ (2%, Table 3, entry 2) of [^11^C]**1A** was observed using 1 equiv. of DBU with [^11^C]dibenzylurea as a major by-product. When the reaction was carried out at a lower amine concentration, a slight increase in RCY was observed (5%, Table 3, entry 3). Interestingly, increasing the DBU concentration further to 1, 2.5, 5 and 10 equiv. led to a RCY of 10% (Table 3, entries 3–6).

We have previously reported that the RCY of [^11^C]ureas is dependent on reaction temperature.^5^ A similar trend was observed for the synthesis of [^11^C]amides. Indeed, varying the temperature from 20 to 30, 40, 50 and 80 °C improved the RCY (Table 3, entries 6–13). The RCY increased from 9% at 20 °C to 27% at 80 °C (Table 3, entries 6–10, Fig. S5, ESI†). Similarly by adding 2 equiv. of Mitsunobu reagents the RCY increased from 10% at 20 °C to 45% at 80 °C (Table 3, entries 11–13, Fig. S5, ESI†).

Interestingly, the increase of Mitsunobu reagents from 1 to 2, 4 and 8 equiv. (Table 3, entries 10 and 13–15), significantly increased the RCY from 27% to 50%. Increasing DBU concentration from 10 to 30 equivalents did not improve the RCY further (28–30%, Table 3, entries 16–17). Good RCY's of the desired [^11^C]amide were observed when MeCN was used as a solvent (50%) while DMF was detrimental for the RCY (0%, Table 3, entry 18).

To demonstrate the potential utility of this novel labelling strategy, the endogenous neurotransmitter melatonin, was radiolabeled with carbon-11 (Scheme 3). The incorporation of [^11^C]CO_2_ into [^11^C]melatonin was 36% – determined by radioHPLC 2 min from end of [^11^C]CO_2_ delivery with an estimated molar radioactivity of 70–100 GBq μmol^–1^, consistent with the molar radioactivities obtained for other ^11^C-labelled tracers at our institution.^21^

**Scheme 3:**
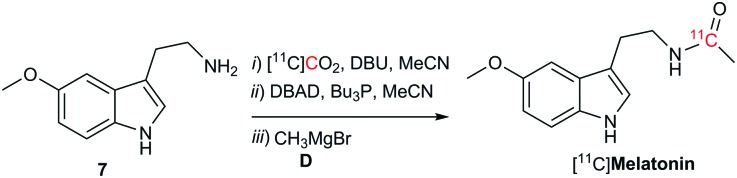
Reaction conditions: [^11^C]CO_2_ was bubbled in a solution of **7** (32 μmol, 1 equiv.), DBU (10 equiv.) in MeCN (160 μL) at room temperature. Then, the reaction mixture was heated (50 °C) for 30 seconds. Mitsunobu reagents (8 equiv.) in MeCN (100 μL) were added and stirred for 10 s. **D** (8 equiv., 3.0 M in Et_2_O) was added. The reaction was quenched after 1 min.

In summary a rapid one-pot methodology for the synthesis of amides has been successfully developed. Readily available CO_2_ was incorporated into primary or activated aromatic amines in the presence of Mitsunobu reagents to form the isocyanate intermediate. Grignard reagents were then reacted with the isocyanate to form the corresponding amides. The approach was optimised for the radiolabelling of [^11^C]amides using [^11^C]CO_2_ directly from the cyclotron. The synthesis time from end of [^11^C]CO_2_ delivery was less than 2 minutes. Radiolabelling of a biologically relevant biomolecule, [^11^C]melatonin, was achieved. A limitation of the proposed technique is the restricted number of commercially available Grignard reagents and their chemical stability. However these reagents can easily be produced in-house upon demand. As some functional groups are not compatible with Grignard reagents this should be borne in mind when using this methodology. In conclusion, this alternative approach offers new possibilities for the rapid and efficient carbon-11 labelling of drugs containing amide functional groups for PET imaging applications.

This work was supported by Medical Research Council (MRC, MR/K022733/1) and European Commission, FP7-PEOPLE-2012-ITN (316882, RADIOMI). The authors acknowledge financial support from the Department of Health *via* the National Institute for Health Research (NIHR) comprehensive Biomedical Research Centre award to Guy's & St Thomas' NHS Foundation Trust in partnership with King's College London and King's College Hospital NHS Foundation Trust and the Centre of Excellence in Medical Engineering funded by the Wellcome Trust and EPSRC under grant number WT 088641/Z/09/Z.

## Supplementary Material

Supplementary information
