# Supplementary material for: From [11C]CO2 to [11C]amides: a rapid one-pot synthesis via the Mitsunobu reaction
Source: Chem Commun (Camb). 2017 Apr 27;53(38):5334–7. doi: 10.1039/c7cc01407d (PMC5708528; doi:10.1039/c7cc01407d)
Supplement: Supplementary file 1 [file CC-053-C7CC01407D-s001.pdf]

Electronic Supplementary Material (ESI) for ChemComm.
This journal is © The Royal Society of Chemistry 2017

Supporting Information

From [^{11}C]CO_2 to [^{11}C]Amides: A Rapid One-Pot Synthesis *via* Mitsunobu Reaction

Salvatore Bongarzone,[a] Anne Runser,[a] Carlotta Taddei,[a] Abdul Karim Haji Dheere[a] and Antony Gee[a]*

[a] Division of Imaging Sciences and Biomedical Engineering King's College London, King's Health Partners, St. Thomas' Hospital London, SE1 7EH, United Kingdom E-mail: antony.gee@kcl.ac.uk

Contents

General Method and Materials…………………………………………………………………… **S1**

Procedure for synthesis of 1A and 4A…………………………………………………………… **S1**

Procedure for synthesis of 1C, 5C and 6C………………………………………………………… **S2**

Carbon-11 Radiochemistry…………………………………………………………………………… **S3**

Radio-HPLC traces for [^{11}C]1A and [^{11}C]Melatonin ……………………………………………… **S5**

Figure S4 and S5……………………………………………………………………………………… **S6**

General Method and Materials

N-Benzylamine (99%), *N*-benzylmethylamine (97%), 3-phenyl-1-propylamine (98%), 2,4,6-trimethylaniline (98%), cyclohexylamine (≥ 99.9%), ethyl acetate (EtOAc, ≥ 99.9%), hexane (≥ 99.9%), 1,8-diazabicyclo[5.4.0]undec-7-ene (DBU, 98%), oxalic acid (≥99%) and silica gel (200 – 425 mesh) were purchased from Sigma-Aldrich. Anhydrous acetonitrile (MeCN, 99.8%), anhydrous diethyl ether (Et_2O, ≥ 99%), ethyl acetate (EtOAc, ≥ 99.9%) and hexane (≥ 99.9%) were purchased from Sigma-Aldrich. Mitsunobu reagents used were di-*tert*-butyl azodicarboxylate (DBAD, ≥ 98.0%, Fluka), diethyl azodicarboxylate (DEAD, 97%, Alfa Aesar), tributylphosphine (PBu_3), triphenylphosphine (PPh_3, 99%, Avocado). Grignard reagents, 1-propynylmagnesium bromide (0.5 M in THF), ethylmagnesium bromide (1.0 M in THF), phenylmagnesium bromide (1.0 M in THF) were purchased from Sigma-Aldrich. Aniline and *N*-methylaniline (≥ 98.0%) were respectively purchased from Acros Organics and Fluka. Carbon dioxide (CO_2) was purchased from BOC GASES (CAS number: 124-38-9). ^{1}H and ^{13}C-NMR spectra were obtained using a BRUKER AVANCE DRX 400 MHz spectrometer. Mass spectroscopy was performed using on an Agilent 6520 Accurate-Mass Q-TOF LC/MS connected to an Agilent 1200 HPLC system with UV detector and autosampler.

Procedure for synthesis of 1A and 4A

The amine (138.6 μmol, 1 equiv.) and DBU (1 μL, 6.9 μmol, 0.05 equiv.) were added in dry MeCN (1 mL) under nitrogen. Carbon dioxide (CO_2) was bubbled into the reaction mixture for 40 min. To this solution was added a solution of DBAD (64.5 mg, 277 μmol, 2 equiv.) and Bu_3P (7.2 μL, 277 μmol, 2 equiv.) in dry MeCN (0.5 mL) prepared under nitrogen and stirred for 10 min. The reaction mixture was stirred for additional 10 min before adding the Grignard reagent (1 mmol, 7.2 equiv.) under nitrogen. After 30 min of magnetic stirring, the reaction was quenched with a solution of oxalic acid (0.6 M, 2 mL). Then the solvent was evaporated and the product extracted with EtOAc washes (3 x 20 mL). The organic phase was washed with brine (20 mL), dried over $MgSO_4$, concentrated under reduced pressure and purified by flash column chromatography (silica gel, 100–70% hexane/EtOAc gradient).

***N*-benzylbut-2-ynamide (1A)**
Yield 38 ± 12%. ^{1}H NMR (400 MHz, $CDCl_3$): δ 7.30 – 7.18 (m, 5H), 4.38 (d, J=5.9, 2H), 1.85 (s, 3H). ^{13}C NMR (100 MHz, $CDCl_3$): δ 174.53, 152.43, 136.43, 136.36, 127.78, 127.73, 126.81, 126.74, 126.66, 126.10, 82.85, 76.38, 76.06, 75.74, 73.65, 46.34, 42.73, 19.80, 3.04, 2.64. HRMS (ESI): Calc. [M + H^+] 174.0919; found: 174.0967.

***N*-(2,4,6-trimethylphenyl)but-2-ynamide (4A)**
Yield 37%. ^{1}H-NMR (400 MHz, $CDCl_3$) δ 6.82 (s, 2H), 2.19 (s, 3H), 2.14 (s, 6H), 1.94 (s, 3H). ^{13}C NMR (100 MHz, $CDCl_3$) δ 151.93, 137.59, 135.30, 129.95, 128.99, 84.07, 75.01, 20.95, 18.34, 3.79. HRMS (ESI): Calc. [M + H^+] 202.1232; found: 202.1235.

General procedure for synthesis of 1C, 5C and 6C

The corresponding amine (**1**, **5** or **6**, 138.6 µmol, 1 equiv.) and DBU (1 µL, 6.9 µmol, 0.05 equiv.) were added in dry Et_2O (1 mL) under nitrogen. The sealed vial was placed in an ice-bath and CO_2 was bubbled for 5 min at 0°C. The reaction mixture was stirred for 35 min at room temperature. To this solution was added a solution of DBAD (64.5 mg, 277 µmol, 2 equiv.) and Bu_3P (70 µL, 277 µmol, 2 equiv.) in dry Et_2O (0.5 mL) prepared under nitrogen and stirred for 10 min. The reaction mixture was stirred for additional 10 min before adding phenylmagnesium bromide (**C**, 1 mL, 1 mmol, 7.2 equiv.) at 0 °C under nitrogen. Then the reaction was warmed at room temperature and stirred 30 min before being quenched with oxalic acid (0.6 M, 2 mL). The organic phase was washed with brine (20 mL), dried over $MgSO_4$, concentrated under reduced pressure and purified by flash column chromatography (silica gel, 100–70% hexane/EtOAc gradient).

***N*-benzylbenzamide (1C):** Yield 28%. ^1H NMR (400 MHz, $CDCl_3$) δ 7.74 – 7.69 (m, 2H), 7.44 – 7.18 (m, 8H), 6.49 (s, 1H), 4.55 (d, 2H). ^{13}C NMR (101 MHz, $CDCl_3$) δ 167.4, 138.2, 134.4, 131.6, 128.9, 128.6, 127.9, 127.6, 127.0, 44.1. HRMS (ESI): Calc. [M + H^+] 212.1075; found: 212.1421.

***N*-(3-phenylpropyl)benzamide** (**5C**): yield 19%. ^1H NMR (400 MHz, $CDCl_3$) δ 7.72 – 7.67 (m, 2H), 7.53 – 7.47 (m, 1H), 7.42 (tt, J=6.7, 1.5, 2H), 7.35 – 7.20 (m, 5H), 6.31 (d, J=44.6, 1H), 3.52 (dd, J=13.7, 7.2, 2H), 2.79 – 2.70 (m, 2H), 1.99 (dt, J=13.7, 7.2, 2H). ^{13}C NMR (100 MHz, $CDCl_3$) δ 167.5, 141.5, 134.6, 131.4, 128.6, 128.5, 128.4, 126.8, 126.1, 39.8, 33.5, 31.1. HRMS (ESI): Calc. [M + H^+] 240.1388; found: 240.1402.

***N*-cyclohexylbenzamide** (**6C**): yield 5%. ^1H NMR (400 MHz, $CDCl_3$) δ 7.70 – 7.66 (m, 2H), 7.44 – 7.38 (m, 1H), 7.35-7.32 (m, 2H), 3.96 – 3.85 (m, 1H), 2.01 – 1.89 (m, 2H), 1.72 – 1.63 (m, 2H), 1.62 – 1.54 (m, 2H), 1.42 – 1.28 (m, 2H), 1.23 – 1.10 (m, 2H). ^{13}C NMR (100 MHz, $CDCl_3$) δ 166.7, 135.1, 131.2, 128.5, 126.8, 48.7, 33.2, 25.5, 24.9. HRMS (ESI): Calc. [M + H^+] 204.1388; found: 204.1613.

1. Carbon-11 Radiochemistry

Materials: *N*-benzylamine (99%), 5-methoxytryptamine. 1,8-diazabicyclo[5.4.0]undec-7-ene (DBU, 98%) and anhydrous acetonitrile (MeCN, 99.8%) were purchased by Sigma-Aldrich. Mitsunobu reagents used are di-*tert*-butyl azodicarboxylate (DBAD, ≥ 98.0%, Fluka), tributylphosphine (Bu_3P). Grignard reagents, 1-propynylmagnesium bromide (0.5 M in THF) and methylmagnesium bromide (3.0 M in diethyl ether), melatonin were purchased from Sigma-Aldrich. A solution of MeCN/H_2O (1:1) was used to quench the reaction. **1A** and melatonin were used as reference compounds. [^{11}C]CO_2 was produced using a Siemens RDS112 cyclotron by the 11 MeV proton bombardment of nitrogen (+ 2% O_2) gas via the $^{14}N(p,\alpha)^{11}C$ reaction. The cyclotron-produced [^{11}C]CO_2 was bubbled in a stream of helium gas with a flow rate of 60 mL/min post target depressurisation directly into a reaction v-vial. HPLC analysis was performed on an Agilent 1200 system equipped with a UV detector (λ=254 nm) and a β^+-flow detector coupled in series.

General Radiosynthetic procedure

An oven-dried vial (KX Microwave Vials, 5 mL) and a crimp cap (Fisherbrand, centre hole with 3.0 mm PTFE seal aluminum silver 20 mm, part # 10132712) were used. All gas transfer lines were fabricated from PTFE tubing (length: 10–30 cm, O.D.: 0.79 x 0.4 in., I.D.: 1/32 x 0.16 in.). A P_2O_5 trap and one-way valve (BRAUN, normally closed backcheck valve, part # 415062) were placed before the vial. An ascarite® trap consisting of a cartridge (Biosys Solutions Ltd, Fritted Empty MiniSpeed Cartridges, part # 2447) filled with ascarite (Sigma-Aldrich, 1310-73-2) was placed after the Vial to trap unreacted [^{11}C]CO_2. A waste bag was placed at the outlet to prevent any gaseous emission. A cyclotron beam current of 5 µA was maintained for a bombardment time of 1 min for all reaction optimization experiments producing ~ 300 MBq of carbon-11. The cyclotron-produced [^{11}C]CO_2 was bubbled through a reaction vial containing the amine, DBU and anhydrous acetonitrile in a stream of helium gas with a flow rate of 50-60 mL/min post target depressurisation. The vial was placed in a heating block and the reaction mixture was heated for 30 seconds. Mitsunobu reagents in CH_3CN (100 µL) were added and stirred for 10 sec. Grignard reagent (8 equiv.) was added (NOTE: To avoid over-pressure of gases inside the vial upon addition of Grignard reagents, a waste bag was attached to the vial *via* a vent needle). The reaction was quenched after 1 min with a solution of H_2O/MeCN (200 µL). An aliquote of the crude was injected in the radio-HPCL (C18 column, UV and radio detector) in order to determine the RCY. Analytical reverse-phase column (ZORBAX Eclipse XDB-C18, 4.6 x 150 mm, 5 µm) was used with a flow rate of 1 mL/min. The gradient was linear between 20–80% over 5 min (MeCN:H_2O, 20:80), isocratic between 5–7 min (MeCN:H_2O, 80:20) and isocratic between 8–11 min (MeCN:H_2O, 20:80). Semi-preparative reverse-phase column (Jupiter, 10 x 150 mm, 5 µm) was used with a flow rate of 1.5 mL/min to purify [^{11}C]Melatonin crude reaction. The method was isocratic over 17 min (MeCN:H_2O, 30:70), gradient between 17–20 min (MeCN:H_2O, 90:10) and isocratic between 20–25 min (MeCN:H_2O, 30:70). Identification of all radioactive products was confirmed by co-elution with the corresponding non-radioactive compounds.

Radio-HPLC traces for [^{11}C]**1A** and [^{11}C]Melatonin

[^{11}C]Melatonin was purified by preparative HPLC using a reverse-phase column (Jupiter, 10 x 150 mm, 5 µm) with a flow rate of 1.5 mL/min (70% water and 30% Acetonitrile, isocratic). The retention time of Melatonin is 9 minutes and 38 seconds. Radiochemical purity was assessed by injecting the radiotracer alone. Identity of the radiolabelled product was confirmed by co-injection with the unlabelled reference standard (see chromatograms below).

Fig. SI1 A) Radio-HPLC chromatogram of crude [^{11}C]**1A**. **B)** UV chromatogram of crude [^{11}C]**1A**. **C)** UV chromatogram of crude [^{11}C]**1A** co-injected with **1A**. The difference between UV peaks (retention time (t_R) = 5 minutes and 8 seconds) and radioactivity peaks (t_R = 5 minutes and 27 seconds) is 19 seconds and is in the range of delay of this instrument (~20 seconds).

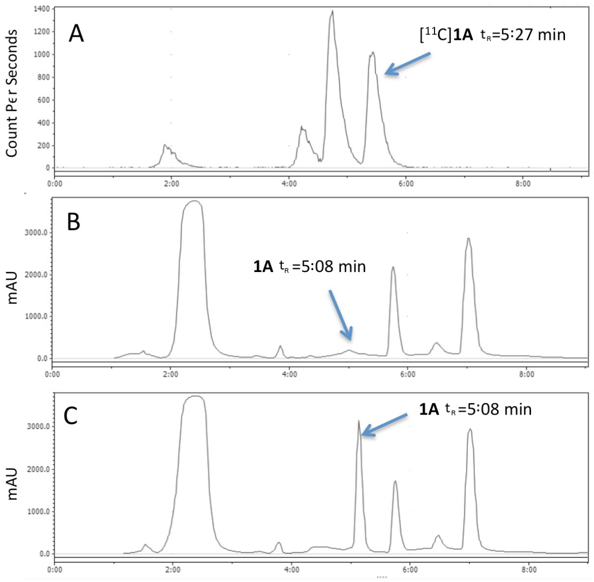

Fig. S2 **A)** Radio-HPLC radiochromatogram of purified [11C]Melatonin. **B)** UV-Chromatogram of purified [11C]melatonin spiked with non-radioactive Melatonin. The difference between UV peaks (retention time (t_R) = 3 minutes and 58 seconds) and radioactivity peaks (t_R = 4 minutes and 16 seconds) is 18 seconds and is in the range of delay of this instrument (~20 seconds).

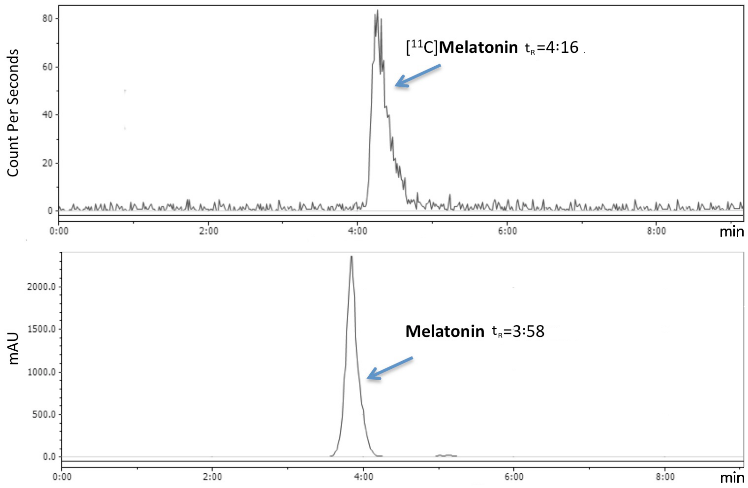

Fig. S3 Calibration Curve for Melatonin.

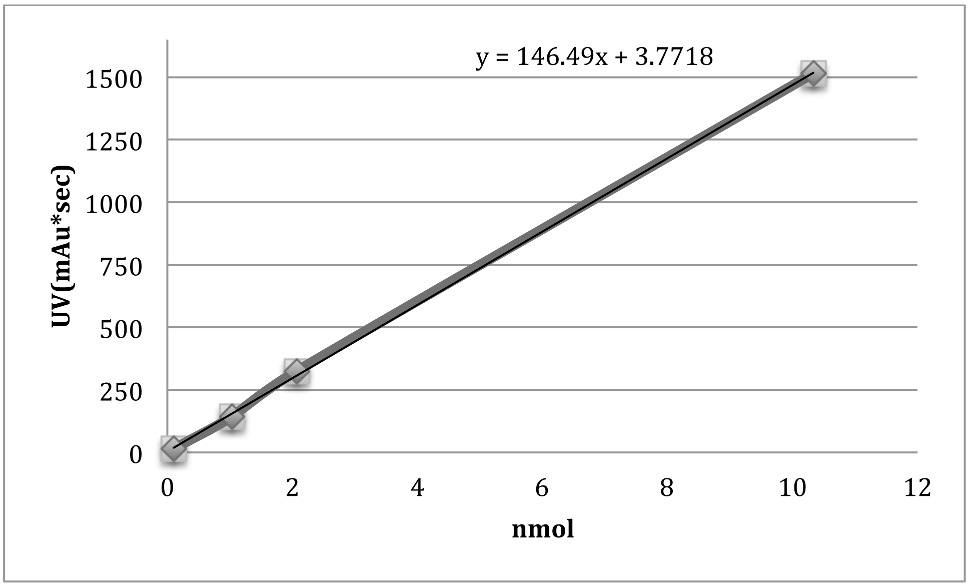

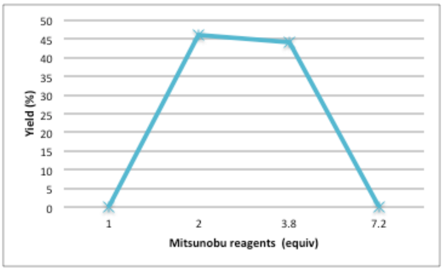

Fig. S4 Graphical representation of entries 5-7 reported in Table 1.

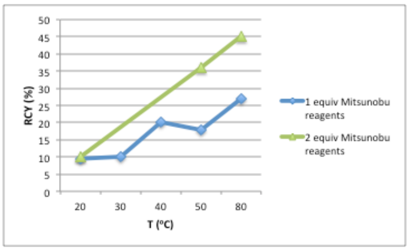

Fig. S5 Graphical representation of entries 6-14 reported in Table 3.
